# Cardiovascular Risk in Fatty Liver Disease: The Liver-Heart Axis—Literature Review

**DOI:** 10.3389/fmed.2019.00202

**Published:** 2019-09-13

**Authors:** Abdulrahman Ismaiel, Dan L. Dumitraşcu

**Affiliations:** ^1^Iuliu Haţieganu University of Medicine and Pharmacy, Cluj-Napoca, Romania; ^2^2nd Department of Internal Medicine, Cluj-Napoca, Romania

**Keywords:** cardiovascular disease (CV disease), non-alcoholic fatty liver disease (NAFLD), alcoholic liver disease (ALD), cardiac arrhythmias, metabolic syndrome (MetS)

## Abstract

According to the World Health Organization, cardiovascular disease (CVD) remains the leading cause of death worldwide, accounting for approximately 18 million deaths per year. Nevertheless, the worldwide prevalence of metabolic diseases, such as type 2 diabetes mellitus, obesity, and non-alcoholic fatty liver disease (NAFLD), also known to be common risk factors for CVD, have dramatically increased over the last decades. Chronic alcohol consumption is a major cause of chronic liver diseases (CLD) as well as being a major health care cost expenditure accounting for the spending of tremendous amounts of money annually. NAFLD has become one of the major diseases plaguing the world while standing as the most common cause of liver disease in the Western countries by representing about 75% of all CLD. Currently, the most common cause of death in NAFLD remains to be CVD. Several mechanisms have been suggested to be responsible for associating FLD with CVD through several mechanisms including low-grade systemic inflammation, oxidative stress, adipokines, endoplasmic reticulum stress, lipotoxicity and microbiota dysbiosis which may also be influenced by other factors such as genetic and epigenetic variations. Despite of all this evidence, the exact mechanisms of how FLD can causally contribute to CVD are not fully elucidated and much remains unknown. Moreover, the current literature supports the increasing evidence associating FLD with several cardiovascular (CV) adverse events including coronary artery disease, increased subclinical atherosclerosis risk, structural alterations mainly left ventricular hypertrophy, increased epicardial fat thickness, valvular calcifications including aortic valve sclerosis and mitral annular calcification and functional cardiac modifications mainly diastolic dysfunction in addition to cardiac arrhythmias such as atrial fibrillation and ventricular arrythmias and conduction defects including atrioventricular blocks and bundle branch blocks. Patients with FLD should be evaluated and managed accordingly in order to prevent further complications. Possible management methods include non-pharmacological strategies including life style modifications, pharmacological therapies as well as surgical management. This review aims to summarize the current state of knowledge regarding the pathophysiological mechanisms linking FLD with an increased CV risk, in addition to associated CV adverse events and current management modalities.

## Key Concepts

- Both alcoholic and non-alcoholic fatty liver diseases are associated with intrahepatic and extrahepatic manifestations such as fatal and non-fatal cardiovascular diseases, leading to an increased morbidity and mortality.
- Features of the MetS are frequently present in non-alcoholic fatty liver disease (NAFLD) patients associating them with a higher CV risk.
- Several factors have been described as possible pathogenic factors playing a role in the explanation of the complex relationship associating CVD with FLD such as insulin resistance, systemic inflammation, cytokines, oxidative stress, adipokines, hepatokines, genes, and intestinal microbiota, as well as several other factors.
- Although the current evidence supports the association between FLD and an increased cardiovascular risk, the presence of a causal association remains to be proved.
- Further research is required in order to confirm the causal pathogenic links associating FLD with an increased cardiovascular risk.
- Current convincing evidence describes the relationship associating fatty liver disease (FLD) with an increased cardiovascular risk described by subclinical atherosclerosis in addition to cardiac structural and functional abnormalities.
- Subclinical atherosclerosis demonstrated to be related to FLD consists of coronary artery disease as well as increased carotid intima media thickness and the presence of carotid plaques.
- Structural cardiac alterations associated with FLD include left ventricular hypertrophy, increased epicardial fat thickness, valvular calcifications including aortic valve sclerosis and mitral annular calcification.
- Functional cardiac abnormalities linked to FLD include cardiac arrhythmias of both atrial and ventricular origin in addition to conduction defects including atrioventricular blocks and bundle branch blocks.
- A multidisciplinary patient centered and personalized medicine approach is required to provide FLD patients with the best management.
- Lifestyle changes such as weight loss, exercise and dietary changes are considered the cornerstone in managing FLD patients.
- Possible pharmacological therapies include anti-diabetic drugs such as metformin, SGLT2 inhibitors, PPARγ and GLP-1 agonists, lipid lowering medications mainly statins as well as renin angiotensin system inhibitors.
- Surgical management remains of limited use due to its invasiveness, high costs and possible complications.

## Introduction

According to the World Health Organization, the most common cause of death worldwide is cardiovascular disease (CVD), accounting for an estimated 18 million deaths per year (1). Nevertheless, a dramatic worldwide increase in the prevalence of metabolic diseases such as non-alcoholic fatty liver disease (NAFLD), type 2 diabetes mellitus and obesity, that are known risk factors for CVD, has been observed.

Chronic liver diseases (CLD) contribute for a major burden to both morbidity and mortality worldwide with a significant impact on public health. Fatty liver disease (FLD) is composed of a wide spectrum including alcoholic liver disease (ALD) and non-alcoholic fatty liver disease (NAFLD). Currently, ALD is the second most common indication for liver transplantation for CLD in the Western world. Nevertheless, NAFLD is expected to become the main cause for liver transplantation within 10 years (2).

Chronic alcohol consumption is a major cause of CLD as well as being a major health care cost expenditure accounting for the spending of tremendous amounts of money annually (3). NAFLD has become one of the major diseases plaguing the world while standing as the most common cause of liver disease in the Western countries by representing approximately 75% of the chronic liver disease (4). A recent meta-analysis with a total number of 8,515,431 patients reported that the global prevalence of NAFLD is 25.24% with highest prevalence in the Middle East and South America and lowest in Africa (5). In Romania, the prevalence of NAFLD was found to be 20% of 3,005 hospitalized patients not known to have liver disease (6).

Several studies demonstrated the presence of a direct association between NAFLD and CVD suggesting that NALFD should be considered a significant independent risk factor in the absence of traditional cardiovascular (CV) risk factors and metabolic syndrome for subclinical and clinical CVD (7, 8). Alcoholic hyperlipidemia, associated with its thrombogenic, proinflammatory and pro-coagulative effects, is a definite CV risk factor in drinkers (9).

The current literature demonstrated an association between NAFLD and CV complications such as coronary arteries disease (CAD), subclinical atherosclerosis, and cardiac arrythmias as well as conduction, structural and functional alterations (10). This additional CVD related to NAFLD can further contribute to an increased CV morbidity and mortality (11). The pathogenic factors associated with fatty liver disease are multifactorial (12). These include inflammation, adipokines, intestinal dysbiosis, genetics, oxidative stress as well as psychological stress such as anxiety and depression (13) among others which are all established markers for CVD. This indicates the need for further research and measures to be undertaken in order to reduce the burden caused by FLD.

In this review, we summarize the current NAFLD and ALD definitions in addition to general considerations correlating FLD with CVD. We also discuss possible risk factors and pathophysiological mechanisms that might be associating FLD with CVD. Moreover, we also focus on the updates recently published in the expeditiously increasing clinical evidence associating FLD with CAD, subclinical atherosclerosis risk, structural and functional cardiac modifications in addition to cardiac arrhythmias and conduction defects. Lastly, we will be discussing current updates related to possible management modalities. We believe that this review provides health-care professionals with an update on CVD in patients with FLD in order to provide patients with the required CV screening and evaluation methods, in addition to management strategies.

## Non-Alcoholic Fatty Liver Disease and Alcoholic Liver Disease

### Non-alcoholic Fatty Liver Disease

NAFLD is defined by imagistic or histological presence of hepatic steatosis and the absence of other secondary causes of hepatic fat accumulation such as significant alcohol consumption and other causes mentioned in Table 1 (12). Histologically, NAFLD is considered present when ≥5% of the liver cells contain fat and is considered severe when ≥30% of liver cells contain fat on liver biopsy (14, 15). The term NAFLD includes the entire spectrum of FLD as described in Table 2 according to the American Association for the Study of Liver Diseases (16). The spectrum includes non-alcoholic fatty liver (NAFL), non-alcoholic steatohepatitis (NASH) and liver cirrhosis.

**Table 1:** Common causes of secondary hepatic steatosis.

**MACROVESICULAR STEATOSIS**
• Significant alcohol consumption
• Hepatitis C (especially genotype 3)
• Wilson's Disease
• Lipodystrophy
• Starvation
• Parenteral nutrition
• Abetalipoproteinemia
• Medications (e.g., amiodarone, methotrexate, tamoxifen, corticosteroids, mipomersen, lomitapide)
**MICROVESICULAR STEATOSIS**
• Reye's syndrome
• Acute fatty liver of pregnancy
• HELLP syndrome
• Metabolic disorders (e.g., lecithin-cholesterol-acyltransferase (LCAT) deficiency, cholesterol ester storage disease, Wolman's disease)
• Medications (e.g., valproate, antiretroviral drugs)


**Table 2:** Non-alcoholic fatty liver disease spectrum and definitions.

**NAFLD Spectrum**	**Definition**
Non-alcoholic fatty liver	The presence of ≥5% hepatic steatosis in the absence of hepatocellular injury manifested by hepatocyte ballooning. It is associated with a minimal progression risk of cirrhosis and liver failure.
Non-alcoholic steatohepatitis	The presence of ≥5% hepatic steatosis and inflammation with hepatocyte injury manifested by hepatocyte ballooning, in the presence or absence of liver fibrosis. It is associated with a progression risk to liver cirrhosis, liver failure and can rarely lead to liver cancer.
Non-alcoholic steatohepatitis cirrhosis	The presence of liver cirrhosis with previous or current histological evidence of steatosis or steatohepatitis.
Cryptogenic cirrhosis	The presence of liver cirrhosis without any obvious etiology. It is most commonly associated with metabolic risk factors such as metabolic syndrome and obesity.

### Alcoholic Liver Disease

ALD is defined by imagistic or histological presence of hepatic steatosis associated with significant alcohol consumption of ≥20 grams per day for women and ≥30 grams per day for men; 10 grams of alcohol is equivalent to 4 ounces of wine, 1.5 ounces of hard liquor and 12 ounces of beer (17). The term ALD includes the entire spectrum of FLD as defined in Table 3 starting from alcoholic fatty liver (AFL), alcoholic steatohepatits (ASH) and reaching to alcoholic liver cirrhosis (18, 19).

**Table 3:** Alcoholic liver disease spectrum and definitions.

**ALD Spectrum**	**Definitions**
Alcoholic fatty liver	Increased hepatic fat deposition due to significant alcohol consumption. The average weight for a healthy liver is 1.5 kg and is heavier in patients with alcoholic fatty liver reaching to about 2.0–2.5 kg.
Alcoholic steatohepatitis	A form of toxic liver disease due to chronic excessive alcohol consumption. It is defined histologically by the presence of steatosis, necrosis, inflammation with infiltration of polymorphic granulocytes and fibrosis associated with intracellular Mallory-Denk bodies.
Alcoholic liver cirrhosis	It is defined by a continuous deposition of extracellular matrix and fibrosis associated with the generation of regenerative nodules, in addition to a parallel destruction of the hepatic lobule's normal structure. It represents the irreversible end stage of alcoholic liver disease.

## Fatty Liver Disease, Metabolic Syndrome and Type 2 Diabetes

Metabolic syndrome (MetS) is an important risk factor that identifies subjects with an increased susceptibility of developing future type 2 diabetes and CVD. It is defined by the presence of three of the five criteria mentioned in Table 4 (20). Features of the MetS are frequently met in patients with NAFLD linking it to an increase CV risk (21). Almost 90% of patients with NAFLD will present with at least one of the features of MetS and about 33% will fulfill the criteria for diagnosing MetS (22). A recent study that took place in 19 European centers demonstrated a significant association between decreased insulin sensitivity and an increased incidence of CVD in patients with NAFLD (23). Rocha et al. conducted a study on NAFLD patients demonstrating that 98% of these patients were insulin resistant which is considered a landmark of MetS, while 39% of these patients were diabetic in addition to a marked increase in body mass index (BMI) and waist/hip ratio (24). These results were also confirmed by a recently published meta-analysis demonstrating an association between NAFLD with insulin resistance and diabetes (25). They also added that NAFLD predicted the development of diabetes. While type 2 diabetes mellitus can predict the development of NAFLD and vice versa and each factor can act as a progression factor for the other, the exact association between them remains poorly understood. Moreover, wide spectrums of liver diseases related to NAFLD are associated with obesity such as hepatic steatosis, fibrosis and cirrhosis (26).

**Table 4:** Metabolic syndrome criteria^*^.

**Criteria**	**Definition**
Waist circumference	≥102 cm (40 in) in men or ≥88 cm (35 in) in women
Triglyceride level	≥150 mg/dL (or receiving drug therapy for hypertriglyceridemia)
Blood pressure	≥130/85 mmHg (or receiving drug therapy for hypertension)
HDL cholesterol	< 40 mg/dL in men or < 50 mg/dL in women (or receiving drug therapy for reduced HDL cholesterol)
Fasting blood sugar	≥100 mg/dL (or receiving drug therapy for hyperglycemia)

* *Metabolic Syndrome is diagnosed when an individual has at least 3 of the 5 previously mentioned criteria*.

On the other hand, the protective association of alcohol consumption in CVD remains elusive and inconsistent. The current literature describing ALD has demonstrated an association between alcohol intake and incident type 2 diabetes mellitus (27) or frank type 2 diabetes mellitus due to chronic pancreatitis (28). Moreover, several studies demonstrated that alcohol consumption is associated with an increased risk of cancers and intracerebral hemorrhage, especially in elderly subjects (29–31). Certainly, the relationship between alcohol consumption and obesity in the risk of advanced liver disease and cirrhosis is complex. This is because moderate consumption of alcohol may improve insulin sensitivity and other metabolic parameters which are factors that might reduce the development of NAFLD, whereas heavier alcohol consumption may be additive with NAFLD in the risk of progression to cirrhosis. Several important modifiers related to cardiac effects of alcohol are possibly involved in this mechanistic correlation including drinking patterns, type of consumed alcohol, socio-economic status, education, and dietary patterns. Alcohol consumption might have different outcomes according to several factors including the patients' associated comorbidities. Therefore, an assessment of non-cardiovascular effects associated with alcohol consumption should also be performed when evaluating the potential CV risks and benefits associated with alcohol use.

## Pathophysiological Mechanisms of Cardiovascular Disease in Fatty Liver Disease

**Figure 1:**
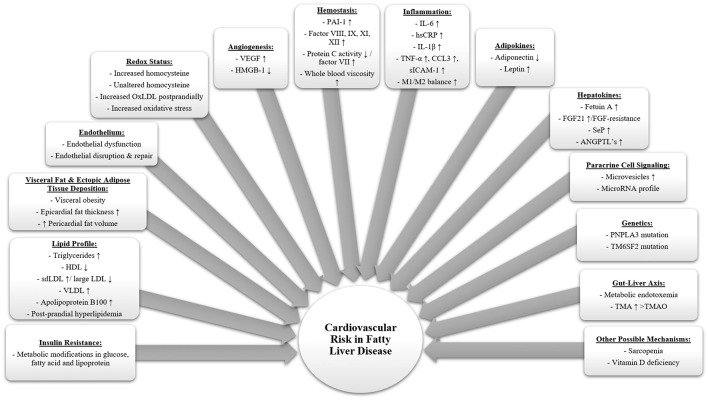
Summary of potential pathophysiological mechanisms associating fatty liver disease with an increased cardiovascular risk. Fatty liver disease leads to multiple alterations in the human body as summarized in this figure, ultimately causing several complications including cardiovascular disease. Low grade systemic inflammation plays an essential role in the development of fatty liver disease. It can be explained by several intertwined factors such as diet, gut, microbiota, genes, visceral adipose tissue and liver. (ANGPTL, Angiopoietin like proteins; FGF21, Fibroblast growth factor 21; HDL, High-density lipoproteins; HMGB-1, High mobility group box 1; hsCRP, High sensitivity C-reactive protein; IL-1b, Interleukin 1b; IL-6, Interleukin 6; LDL, Low density lipoprotein; M1/M2, Macrophage phenotype 1/2 ratio; OxLDL, Oxidized low-density lipoprotein; PAI-1, Plasminogen activator inhibitor 1; PNPLA3, Patatin-like phospholipase domain containing protein 3; sdLDL, Small dense low-density lipoproteins; SeP, Selenoprotein P; sICAM-1, Soluble intercellular adhesion molecule-1; TM6SF2, Transmembrane 6 superfamily member 2; TMA, Trimethylamine; TMAO, Trimethylamine-N-Oxide; TNFα, Tumor necrosis factor a; VEGF, Vascular endothelial growth factor; VLDL, Very low-density lipoprotein).

The complex relationship between FLD and CVD and the mechanistic associations are not yet fully understood which requires further research in this field. Several factors have been described as possible pathogenic causes playing a role in the explanation of this complex relationship associating CVD with FLD as described in Figure 1. Metabolic disorders such as FLD and type 2 diabetes mellitus are known to cause a low-grade systemic inflammation leading to several extrahepatic complications including an increased CV risk (32). Several mechanisms have been suggested to stand behind the presence of this systemic inflammation including oxidative stress, endoplasmic reticulum stress, lipotoxicity and gut microbiota alterations. These mechanisms may also be influenced by other factors such as genetic and epigenetic variations (33–36).

### Inflammation, Cytokines, and Oxidative Stress

The liver is an essential metabolic organ taking part in the systemic inflammation possibly through secreting inflammatory markers, chemokines and cytokines being drained into the systemic circulation, leading to secondary CV adverse through endothelial dysfunction, enhanced plaque formation, altered vascular tone and coagulation. Several inflammatory markers and cytokines have been demonstrated to be related to FLD and an increased CV risk such as serum levels of tumor necrosis factor (TNF)-α, interleukin (IL)-6, CCL3, soluble intercellular adhesion molecule-1 (sICAM-1) and C-reactive protein (CRP) as well as elevated hepatic expression of IL-6, TNF, CXCL10 and IL1RN (37, 38). Worsening inflammatory and insulin resistance (IR) states are associated with worse cardiometabolic outcomes seen in increasing severity of NAFLD.

Oxidation is an essential process for the human body to fight off pathogens, while oxidative stress, a marker of NAFLD, is due to an alteration of the free radical activity and antioxidant activity. This imbalance leads to multiple diseases because of the abnormal release of cytokines such as TNF-α, CRP, and IL-6. Studies suggest that oxidative stress may be involved in the pathogenesis of CVD in patients with FLD through these processes (39) while contributing to the progression of liver disease severity from simple steatosis to steatohepatitis (40).

### Insulin Resistance

IR described as an abnormal cell response to insulin hormone, is one of the principal risk factors demonstrated to be associated with NAFLD, MetS and atherosclerosis. It is suggested that IR evolution might be the principal factor associated with the initiation NAFLD, mainly due to metabolic modifications in glucose, fatty acid and lipoprotein (37). Moreover, hyperinsulinemia was demonstrated to cause an altered cardiometabolic cascade by worsening IR and abnormal insulin signaling in involved tissues (41). The presence of subclinical inflammation, altered adipokines and the presence of increased ectopic fat accumulation in organs can further provoke IR being associated with an increased risk of adverse CV events.

### Dislipidemia and Postprandial Hyperlipidemia

The liver is the main organ in the body responsible for regulating the lipid metabolism process (42). The liver tends to decrease the liver fat by performing a limited compensatory mechanism through increasing liver production of triglyceride (TG) rich very low-density lipoprotein (VLDL) (43) which leads to a reduction in high-density lipoprotein (HDL) levels due to an imbalance in the HDL metabolism. Serum lipid profile is significantly related to the severity of NAFLD demonstrating more alterations in NASH. NAFLD is characterized by an atherogenic lipid profile consisting of high TG levels, low HDL cholesterol, high low-density lipoprotein (LDL) levels, high VLDL levels and high apolipoprotein B100 concentration leading to a significantly increased CV risk (44).

FLD patients can present with postprandial hyperlipidemia defined by elevated TG-rich chylomicron remnants postprandially in addition to a prolonged hypertriglyceridemia which was demonstrated to be associated with postprandial atherogenesis and an increased CV risk (45–47).

### Adipokines

Adipokines are hormones produced by fat tissue influencing the CV system through exerting a direct effect on the vascular wall via paracrine action or affecting endothelial function through an imbalance of plasma and tissue levels of adipokines. Alterations in circulating adipokines such as decreased adiponectin or increased leptin levels were reported in MetS and NAFLD (48). Several adipokines have been demonstrated to present with anti-inflammatory and cardioprotective effects such as omentin, apelin, adiponectin. On the other hand, leptin, visfatin, resistin and adipocyte fatty-acid-binding protein were found to have pro-inflammatory effects exerting a negative impact on the CV function increasing the risk of CVD (49).

### Gut Microbiota

Several factors can modify the enormously complex, dynamic, and vast gut microbiota present in the human body including FLD, diabetes and obesity, leading to adverse CV outcomes (50). Several studies demonstrated that metabolic endotoxemia caused by lipopolysaccharide (LPS) exposure and the binding to toll-like receptor 4 (TLRs) can promote a systemic low-grade inflammation and activate an immune response leading to the release of pro-inflammatory markers (51). This process promotes endothelial dysfunction, oxidation of LDLs and thrombogenesis, in addition to the formation and rupture of atherosclerotic plaques (51). Several novel metabolites studied through metabolomic analyses were found to be associated with an increased CV risk such as phosphatidylcholine (PC) metabolism including choline, betaine, and trimethylamine N-oxide (TMAO) (52). Moreover, TMAO levels were demonstrated to able to predict major adverse cardiac events independently from traditional CV risk factors (53).

### Genetics

A couple of genes seem to be of interest in relation to FLD and CV risk including patatin-like phospholipase domain containing protein 3 (PNPLA3) and transmembrane 6 superfamily member 2 (TM6SF2). PNPLA3 rs738409 mutation was found to be related to the severity of NAFLD and TG metabolism leading to lower plasma lipids, while causing a modest, negative association between PNPLA3 and CAD (38, 54). TM6SF2 may predispose to NAFLD and liver fibrosis because of its role leading to triglycerides and cholesterol retention in the liver. Surprisingly with this mutation, a cardioprotective role described as the “Catch-22” paradigm has been demonstrated due to reduced levels of VLDL secretion and improved serum TG levels with an unaltered insulin sensitivity (38).

### Visceral Fat and Ectopic Adipose Tissue Distribution

Visceral adipose tissue (VAT) is a hormonally active endocrine organ associated with unique biochemical characteristics. It secretes pro-inflammatory cytokines, adipokines and hormones that influence several normal and pathological processes including inflammation and IR (55). The presence of an increased amount of visceral adipose tissue deposition is known as visceral obesity, also known to be associated with several pathologies and risk factors such as MetS and CVD. Studies demonstrated an independent association linking increased liver fat with VAT (56) leading to an increased CV risk, recently assessed and found to be related to plaque calcifications presence and increased CVD (57). Moreover, central obesity is independently associated with an increased cardiovascular morbidity and mortality (58). Furthermore, several known CV risk factors including impaired glucose tolerance, IR and dyslipidemia were demonstrated to be independently associated with an increased VAT mass (59, 60).

Ectopic fat accumulation in the heart possibly evaluated by the presence of an increased epicardial fat thickness (EFT) or increased pericardial fat volume, is demonstrated to be associated with several CV risk factors including visceral fat, IR and MetS leading to an increased risk of adverse CV events such as CAD (37, 61, 62). Moreover, an independent relationship linking NAFLD with ectopic hepatic fat has been studied (63). Furthermore, pericardial fat volume was found to be an independent predictive factor in asymptomatic subjects with major adverse CV events, even after adjusting for coronary artery calcium score, Framingham Risk Score and BMI (64).

## General Considerations Regarding Cardiovascular Disease in Fatty Liver Disease

**Figure 2:**
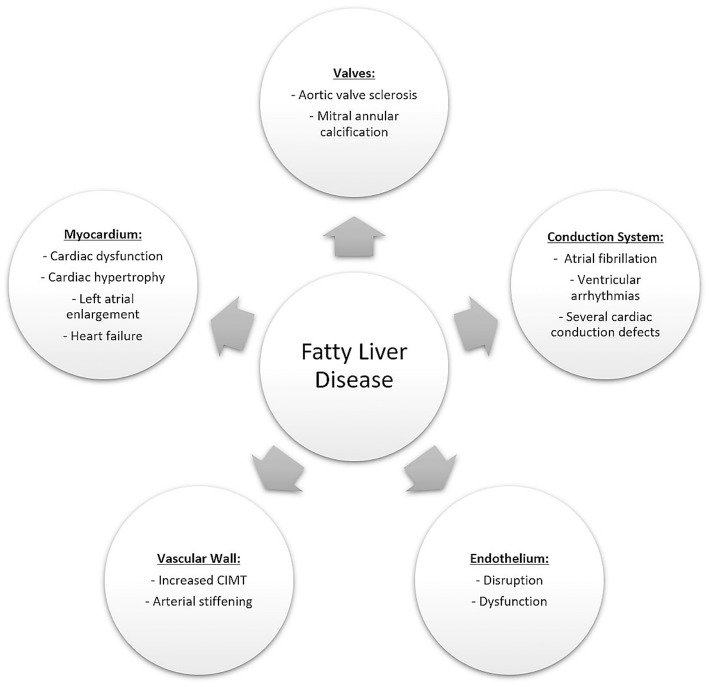
Cardiovascular adverse events associated with fatty liver disease. These can include vascular involvement leading to an increased risk of coronary artery atherosclerosis, ischemic heart disease and an increased carotid intima media thickness. Other complications may include anatomical alterations such as valvular calcifications, functional alterations such as diastolic dysfunction as well as conduction system abnormalities leading to an increased risk of atrial and ventricular arrhythmias.

Several CV complications have been found to be significantly associated with FLD. Figure 2 describes the common CV complications that current studies have demonstrated to be associated with FLD including CAD, subclinical atherosclerotic risk, structural and functional cardiac modifications in addition to cardiac arrhythmias and conduction defects. These CV complications will be further discussed in details in Table 5 and the following sections.

**Table 5:** Cardiovascular complications in fatty liver disease and the common assessment methods.

**Cardiovascular complications in fatty liver disease**	**Common methods of assessment**
Coronary artery disease	Increased coronary artery calcium score—Multiple detector computed tomography
Carotid disease	Increased carotid intima media thickness and presence of carotid plaques—Carotid ultrasound
Structural alterations	Increased left ventricular mass index, interatrial thickness, left atrial stiffness—Transthoracic echocardiography
Epicardial Fat	Increased epicardial fat thickness measurements—Transthoracic echocardiography
Valvular calcifications	Aortic-valve sclerosis and mitral annular calcification—Transthoracic echocardiography
Functional alterations	Diastolic dysfunction—Transthoracic echocardiography
Arrythmias	Atrial fibrillation, ventricular arrhythmias—Electrocardiogram
Conduction alterations	Atrioventricular blocks, bundle branch blocks—Electrocardiogram
QTc interval	Prolonged QTc interval—Electrocardiogram

It is challenging to assess the role that FLD plays in increasing CVD due to the common risk factors present in both pathologies. Several epidemiological studies demonstrated an increased incidence of adverse CV events in patients with FLD in comparison with the general population (7, 37). Söderberg et al. conducted a study with a 28 year follow up for patients with liver biopsy performed due to elevated liver function tests which demonstrated that patients with NAFLD presented with an increased total mortality rate compared to a matched reference population, whereas CVD was the main cause of death, exceeding cancer and liver related mortality (7).

On the other hand in regard to ALD, several studies demonstrated a U- or J- shaped association between moderate alcohol consumption with 1-2 drinks per day for men and 1 per day for women and cardiovascular benefits such as lower incidence of cardiovascular death and non-fatal myocardial infarction (65). However, alcoholic hyperlipidemia, associated with its thrombogenic, pro-inflammatory and pro-coagulative effects, is a definite CV risk factor in drinkers (9, 66). Currently, evidence is not sufficient to support the recommendation of alcohol use as a protective factor for adverse CV events.

## Cardiovascular Disease in Fatty Liver Disease

### Coronary Artery Disease in Fatty Liver Disease

Several studies evaluated the association between FLD and CAD. Most results demonstrated a significantly increased coronary atherosclerotic risk in the presence of FLD. Coronary artery calcification is an independent marker for adverse CV events resulting in a reduction in the vascular compliance, impaired myocardial perfusion and atherosclerotic extent, leading to an increased risk for developing CAD, as well as abnormal vasomotor responses and increased risk of long-term mortality (67, 68). Most published studies in the current literature assessed CAD risk using multiple detector computed tomography and by calculating the coronary artery calcium (CAC) score which is an appropriate method to evaluate asymptomatic subjects with an intermediate CV risk. On the other hand, fatty liver disease was mainly diagnosed using ultrasound.

Chang et al. conducted a recent cross-sectional study assessing the association of CAC score with FLD of alcoholic and non-alcoholic etiology on 105,328 subjects (69). The study demonstrated that both ALD and NAFLD were associated with an increased CAC score. Moreover, they also found that IR index was demonstrated to be a robust and independent predictor of CAC score, even after controlling for traditional CV risk factors, MetS, and C-reactive protein (CRP). Furthermore, a cross-sectional study on 250 subjects from the prospective population of Rancho Bernardo Study involving mainly white adults in suburban Southern California, conducted by Kim et al. demonstrated that although NAFLD was associated strongly with CAC, it was not found to be an independent factor for coronary atherosclerosis in postmenopausal women (70).

Wolff et al. assessed 2,351 subjects from the population-based Rotterdam Study and demonstrated that a proportional increase in liver fat is associated with an increase in EFT as well CAC independently from traditional CV risk factors (71). On the other hand, Kim et al. conducted a cross-sectional study on 2,238 individuals and reported that although CAC was associated with an increase in both EFT and liver fat, EFT was associated with CAC more strongly compared to NAFLD (72).

Moreover, a cross-sectional study conducted by Jacobs et al. involving 250 subjects mentioned that although elderly subjects had a lower prevalence of NAFLD, they still presented with an increased CAC score and visceral adipose tissue (VAT) (73). Lee et al. studied 21,335 subjects enrolled in a screening program demonstrating that NAFLD was more significantly associated with CAC compared to abdominal obesity (74) while another study involving 2,424 individuals from the Coronary Artery Risk Development in Young Adults study by VanWagner et al. suggested that obesity attenuates the association between NAFLD and subclinical atherosclerosis (75).

Another cross-sectional study aiming to examine the relationship between liver fat and serum alanine transaminase (ALT) with coronary calcification in 1,218 subjects conducted by Jung et al. mentioned that subjects with both hepatic steatosis and elevated levels of ALT were found to be associated with a higher CAC score (76).

A strong association between NAFLD and prevalence of significant CAD determined by coronary angiography has also been consistently reported, albeit with variable thresholds of significant CAD between studies (37, 77–79). A study conducted by You et al. involving 285 asymptomatic individuals not known to have chronic liver and ischemic heart diseases reported that a higher CAC score was independently associated with liver stiffness values evaluated using transient elastography in patients with NAFLD (80).

### Carotid Disease in Fatty Liver Disease

Carotid intima media thickness (CIMT) is independently associated with FLD, subclinical atherosclerosis, myocardial infarction and stroke (81). CIMT measurement and plaque burden by ultrasound can be used to screen asymptomatic individuals as it is a well-validated and widely accepted screening tool to predict CVD (82, 83).

A meta-analysis including 27 studies concluded that NAFLD was independently associated with subclinical atherosclerosis even after adjusting for traditional risk factors such as age, sex, BMI, smoking, LDL cholesterol, insulin resistance and MetS (84). Furthermore, studies reported that carotid plaques were present more frequently in NAFLD patients (85, 86).

Moreover, Kim et al. studied the association of atherosclerotic disease in FLD in relation to gender differences and concluded that males had a higher prevalence of FLD, carotid plaque and increased CIMT values compared to females (87). Martinez-Alvarado Mdel et al. suggested that IR could be a mediator of metabolic abnormalities and subclinical atherosclerosis in females (88). A recent study conducted on 1,007 postmenopausal women by Li et al. reported that NAFLD was correlated with an elevated arterial stiffness risk in postmenopausal women, independent of the presence of MetS (89).

While Petit et al. reported in their study involving 102 type 2 diabetic patients that hepatic steatosis was not linked with increased risk of CVD (90), a more recent cross-sectional study conducted by Nahandi et al. on 151 subjects demonstrated a strong association between NAFLD and atherosclerosis independent of diabetes mellitus, while NAFLD severity and increased liver function tests showed an effect upon the atherosclerotic severity (91).

The severity of histological features of NAFLD were also found to be associated with increasing CIMT (92, 93). Bhatia et al. in a pre-sub study of WELCOME trial reported that a NAFLD severity improvement assessed using magnetic resonance spectroscopy and hepatic necro-inflammatory marker serum cytokeratin-18 was correlated with a reduced CIMT progression (94). According to Targher et al. the relationship associating NAFLD and subclinical CVD is mediated by visceral fat that was assessed by abdominal computed tomography (95).

Several laboratory tests were suggested to be associating hepatic steatosis with an increased risk for subclinical atherosclerosis. Arnic et al. suggested that gamma-glutamyltransferase (GGT) and ALT may play a predictive value for CIMT in NASH patients (96). McKimmie et al. suggested in the diabetic heart study that the correlation between hepatic steatosis and CVD may just be an epiphenomenon although hepatic steatosis was demonstrated to be correlated with dyslipidemia (97).

On the other hand, several studies demonstrated a relationship between ALD and an increased CIMT (98). Qu et al. conducted a cross-sectional study on 152 subjects and assessed the association between ALD and CIMT (99). They concluded that ALD may not only lead to an increased CIMT, but it can also promote the premature occurrence of CIMT thickening. Moreover, a recent cross-sectional study conducted on 160 subjects suggested a “double hit phenomenon” as ALD coupled with H. pylori infection can lead to an important CIMT thickening (100).

### Cardiac Structural and Functional Alterations in Fatty Liver Disease

The current literature provides convincing evidence relating FLD with several functional and structural myocardial modifications in the presence or absence of other coexisting features of MetS. Most studies reported a significant correlation associating NAFLD with left ventricular (LV) functional and structural modifications even after adjusting for the commonly associated cardiometabolic risk factors.

In patients with NAFLD without morbid obesity, hypertension and diabetes, the presence of mildly altered LV geometry and early features of LV diastolic dysfunction can be present. The consistent finding of subclinical cardiac dysfunction in an asymptomatic population with NAFLD is not surprising, given that LV dysfunction and LV mass are strongly correlated with IR, as well as subsequent prognosis. Azzam et al. mentioned the existence of a strong positive correlation relating the amount of liver fat with diastolic dysfunction and IR, the only independent parameters found to be associated with NAFLD in their study (101). In patient with diabetes and NAFLD, early features of LV diastolic dysfunction may be detected.

Liver fibrosis assessed histologically was demonstrated to be related to several cardiac parameters assessed by echocardiography. A study assessing the severity of liver fibrosis and cardiac complications conducted on 147 biopsy proven NAFLD patients by Petta et al. reported that several cardiac structural modifications such as diastolic posterior-wall thickness, LV mass, relative wall thickness, left atrial volume as well as LV diastolic dysfunction, ejection fraction, lower lateral tissue doppler imaging peak early diastolic mitral annulus velocity (E') and the ratio of peak velocity blood flow from gravity in early diastole (E wave) to peak velocity flow in late diastole caused by atrial contraction (A wave) also known as the E/A ratio were all associated with severe liver fibrosis (102). Histological liver severity was also demonstrated to be the only independent predictor of impaired coronary flow reserve in a cross-sectional study involving 136 individuals reported by Yilmaz et al. evaluating the integrity of the coronary microvascular circulation (103). They concluded that a lower coronary flow reserve was present in NAFLD patients compared to healthy controls, even after adjusting for obesity, traditional CV risk factors and MetS.

A recent study conducted by Mahfouz et al. on 260 individuals suggested that increased interatrial thickness and left atrial stiffness index found in NAFLD patients could explain the cause behind an increased incidence of atrial fibrillation (AF) (104).

Several articles described a significant association between EFT and NAFLD. Oguz et al. conducted a cross-sectional study concluding that EFT and osteoprotegerin level were increased while aortic flow propagation velocity was decreased in patients with NAFLD (105). Another study involving 868 subjects from the PLIC Study conducted by Baraghetti et al. reported that hepatic steatosis and EFT were related to an increased incidence of extra-cardiac plaques (106). A study conducted on 100 biopsy proven NAFLD and 50 age and sex matched control subjects by Sunbul et al. reported that NAFLD was correlated with an increased arterial stiffness assessed using pulse wave velocity and augmentation index reflecting both the severity of liver fibrosis and increased EFT values (107). On the other hand, Psychari et al. reported an observational cross-sectional study on 105 patients demonstrating that EFT was not significantly associated with NAFLD *per se*, but to diabetes and inflammation (108).

Moreover, cardiac structural and functional alterations were also demonstrated in patients with ALD. Patients with alcoholic liver cirrhosis can present with cirrhotic cardiomyopathy representing an alteration in cardiac function in the absence of a clear underlying CV cause. Alcoholic cirrhosis exerts an important influence on the CV system and hemodynamics of the heart leading to an increased heart rate and cardiac output, in addition to a decreased systemic vascular resistance, arterial pressure, and plasma volume expansion. Alcoholic cardiomyopathy is demonstrated to present with dilated LV, increased LV mass and a normal or decreased LV wall thickness in addition to systolic and diastolic dysfunction although it starts primarily as an altered diastolic function without abnormal changes in the systolic function at rest (109, 110).

### Heart Valve Calcification in Fatty Liver Disease

Aortic-valve sclerosis and mitral annular calcification, both associated with an increased risk of cardiac arrhythmias, all cause and CV mortality rates, are frequently found on echocardiography in elderly subjects being present in up to 20% of adults older than 65 years old (111–113).

Several studies demonstrated an association between FLD and calcification of the aortic and mitral heart valves. A cross-sectional study conducted by Markus et al. reported that hepatic steatosis was associated with 32% higher odds for developing aortic valve sclerosis in comparison to subjects without hepatic steatosis after adjusting for high-sensitive C-reactive protein (hs- CRP), serum ferritin levels, and white blood cells (114). Moreover, a study conducted on 180 type 2 diabetics by Bonapace et al. demonstrated for the first time a positive and independent association between NAFLD and aortic valve sclerosis in diabetic patients even after adjusting for age, sex, duration of diabetes, diabetes treatment, BMI, smoking, alcohol consumption, hypertension, dyslipidemia, glycated hemoglobin (HbA1c) and estimated glomerular filtration rate (115). Furthermore, Mantovani et al. reported that NAFLD was found to be an independent predictor of cardiac calcification in both the aortic and mitral valves in patients with type 2 diabetes, even after performing adjustments (116).

Till present, several studies demonstrated an association between NAFLD and the presence of aortic and mitral valves calcification mainly in diabetic patients while this association remains to be assessed in non-diabetics with FLD. Moreover, the association between ALD and heart valve calcification remains to be studied.

### Cardiac Arrythmias and Conduction Abnormalities in Fatty Liver Disease

The association between FLD and risk of cardiac arrhythmias, as well as related conduction abnormalities has attracted scientific interest lately. Multiple studies assessed the relationship between FLD with several electrocardiogram findings and demonstrated the presence of an increased risk of AF, prolonged QTc interval, bundle branch and atrioventricular blocks. Moreover, diabetic patients with NAFLD were also demonstrated to present with an increased risk for developing ventricular arrhythmias.

The most common type of sustained arrhythmia is AF being a major health problem due to increased morbidity and mortality. Multiple studies assessed the relationship between FLD and AF. A recent meta-analysis that evaluated 9 cross-sectional and longitudinal studies with a total population of 364,919 subjects reported that NAFLD was correlated with an increased risk of AF in middle-aged and elderly subjects and especially in type 2 diabetics (117). Zhang et al. conducted a cross-sectional study on 1,688 subjects, demonstrating that elderly subjects with NAFLD presented with a significant prevalence of AF, even after adjusting for age, gender, systolic blood pressure, fasting plasma glucose, GGT, HDL cholesterol, triglycerides, total cholesterol and albumin (118). On the other hand, Markus et al. performed a population-based Study of Health in Pomerania on 3,090 subjects and concluded that moderate elevation in serum liver enzymes and not hepatic steatosis diagnosed using ultrasound were correlated with higher AF prevalence rates (119). They suggested that a possible pathogenic mechanism behind this correlation might be attributed to elevated levels of pro-inflammatory, procoagulant and profibronogenic factors associated with increased levels of serum liver enzymes causing structural and electrical alterations of the atrium leading to a higher risk of AF. Moreover, Montovani suggested that hyperuricemia might be playing a mechanistic role in the association between NAFLD and AF (120).

Another retrospective study involving 330 type 2 diabetics conducted by Montovani et al. described a significant association presented for the first time correlating NAFLD with an increased prevalence of >30 PVCs/h, non-sustained ventricular tachycardia or both (121). A 3.5-fold increase risk of ventricular arrhythmias was found in NAFLD patients independent of age, sex, BMI, smoking, hypertension, ischemic heart disease, valvular heart disease, chronic kidney disease, chronic obstructive pulmonary disease, serum GGT levels, medication use, and left ventricular ejection fraction.

Another known risk factor for sudden cardiac death is a prolonged QT interval that was demonstrated to be present in patients with FLD. Hung et al. conducted a cross-sectional analysis on 31,116 subjects demonstrating an increased risk for QTc interval prolongation in both diabetic and non-diabetic patients with NAFLD even after adjusting for known factors associated with the QTc interval (122).

A hospital-based cohort of 751 patients with type 2 diabetes conducted by Montovani et al. demonstrated that subjects with NAFLD presented with a significantly increased prevalence of persistent heart block defined by the presence of at least one block among first-degree atrioventricular block, second-degree atrioventricular block, third-degree atrioventricular block, left bundle branch block, right bundle branch block, left anterior hemi-block or left posterior hemi-block compared to subjects without NAFLD being even stronger among subjects with a higher FIB-4 score (123). Furthermore, a threefold increased risk of prevalent heart block was found to be in NAFLD patients after adjusting for age, sex, hypertension, prior ischemic heart disease, HbA1c, microvascular complication status, use of medications and other potentially confounding factors. Iscen reported that young, healthy subjects with right bundle branch block presented with an increased risk of having NAFLD (124).

Vagal reactivation can be evaluated using the heart rate recovery after performing exercise. Abnormal results present an independent prognostic factor for CV and all-cause mortality. Ozveren et al. reported that NAFLD patients have an altered heart rate recovery index (125).

## Fatty Liver Disease Treatment and Cardiovascular Risk Prevention

**Figure 3:**
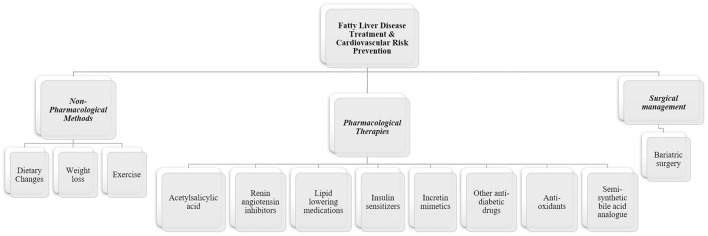
Management strategies in fatty liver disease with cardiovascular risk reduction. Management of fatty liver disease should be individualized to each patient accordingly. Non-pharmacological interventions are essential in the management of fatty liver disease through several lifestyle changes. Currently, no specific pharmacological therapies are approved for NAFLD or NASH. However, several therapeutic drugs were studied and demonstrated positive results in improving hepatic steatosis and liver enzymes as well as decreasing cardiovascular risk, while other drugs are showing promising results in undergoing late phase clinical trials. Surgical management is also possible in specific indicated cases.

Each patient with FLD should be evaluated and managed accordingly. Possible management methods include non-pharmacological strategies, pharmacological therapies as well as surgical management as described in Figure 3.

### Non-pharmacological Methods

Non-pharmacological interventions are the current cornerstone of the management of FLD. Current management of FLD is achieved through several methods including lifestyle changes leading to weight loss and obesity reduction, in addition to the prevention of MetS by controlling cardiometabolic risk factors involved as well as improving NASH in order to prevent the progression of liver fibrosis to cirrhosis and eventually HCC. Studies demonstrated that individualized therapy and lifestyle changes in FLD patients not only improve liver enzyme levels as well as lead to a reduction in hepatic steatosis, injury and fibrosis, but also decrease the risk of developing several complications including CVD, type 2 diabetes mellitus and several types of cancers (16). FLD patients can also decrease their hepatic CV related risk by decreasing their weight and preventing the occurrence of MetS.

#### Dietary Changes

Despite multiple studies discussing different dietary plans recommended for FLD patients, the best dietary plan remains to be known (126). Patients who maintain a long-term calorie restricted diet for 1 year or more were found to have an improvement in hepatic steatosis and decreased CV risk (127, 128). FLD patients are recommended to restrict foods which contain fructose, saturated fats, simple carbohydrates and sugar containing drinks (129) while substituting these with a low fat and high carbohydrate diet in addition to increasing fruits and vegetables intake (130). It has been demonstrated that non-diabetic patients who consume a Mediterranean diet containing high levels of monounsaturated fatty acids presented with an improvement in their hepatic steatosis and insulin sensitivity (131). Furthermore, a recently published meta-analysis mentioned that coffee use may slow down and decrease the risk of liver fibrosis (132). Moreover, alcohol restriction and smoking cessation are essential and also recommended (133).

### Weight Control

Patients with FLD can achieve significant improvement and prevent further liver damage through body weight reduction of 5% or more, regardless of the method used for weight loss such as a low-calorie diet, physical activity or bariatric surgery, with a better outcome in patients loosing 10% or more of their body weight (134, 135). Moreover, several structural and functional cardiac abnormalities seem to be reversible in the presence of a sustained body weight reduction (136–138). Nonetheless, a paradoxical relationship has been demonstrated associating increased BMI with a better prognosis in patients with clinically symptomatic heart failure (139, 140). This can be explained by a potentially cardioprotective effect found in obese patients exerted through a reduced neurohormonal system activation, improved protection toward endotoxin and pro-inflammatory cytokines as well as an increased metabolic and nutritional reserve (140). Accordingly, seeking weight loss in patients with FLD and an associated advanced cardiac dysfunction should be cautiously performed, as well as point out the importance of early stage lifestyle changes in order to prevent complications (16).

#### Exercise

Lack of physical activity is a known risk factor associated with FLD (141, 142). Exercise decreases insulin resistance, a known CV risk factor, through increasing insulin sensitivity at the level of the skeletal muscles, as well as improving hepatic steatosis (143, 144). Moreover, physical activity results in an increased energy expenditure. This can lead to weight loss, as well as decreased intrahepatic triglyceride content (145). Studies demonstrated that resistance, moderate and high intensity training can result in several beneficial hepatic effects such as releasing myokines and other mediators, which act as protective factors in FLD, regardless of weight loss associated with exercise (146). Furthermore, beneficial effects of physical exercise have been demonstrated in FLD patients with CVD (147, 148). Nonetheless, the majority of FLD patients do not succeed to reach their target weight loss goals or to maintain these results for long-term (149).

### Pharmacological Therapies

At the moment, there are no specific approved pharmacological therapies for NAFLD or NASH (150). Despite the absence of firm recommendations for how to manage NASH, several pharmacological therapies are currently available including insulin sensitizers, antioxidants, lipid-lowering agents, incretin-based drugs and weight loss medication (10). Due to the fact that no specific drug is currently approved for treating NAFLD, a quick and rapid evolution in scientific research is under process in order to discover possible pharmacological therapies for treating this pathology.

#### Acetylsalicylic Acid

Despite the controversy regarding the benefits of acetylsalicylic acid **(**ASA) use for primary CVD prevention which was reported in a recent meta-analysis and systematic review including 13 trials and randomizing 164,225 participants demonstrating that subjects without known CVD that received daily aspirin had a lower risk of adverse CV events but with the cost of increasing the risk of major bleeding (151), it is demonstrated to be beneficial and is indicated for secondary prevention in patients with symptomatic atherosclerotic disease. On the other hand, much of the current literature assessing the effect of ASA in FLD has been limited to cross-sectional studies demonstrating that subjects receiving ASA have reduced liver fibrosis indexes levels (152). Moreover, a recent prospective cohort study performed on 361 adults with biopsy-confirmed NAFLD out of which 151 subjects were receiving daily ASA demonstrated for the first time prospectively that daily ASA use in NAFLD was associated with less severe histologic features on biopsy of NAFLD and NASH, in addition to a lower progression risk to advanced fibrosis with the most benefit found in subjects who received daily ASA for ≥4 years (153). Therefore, due to all the robust literature demonstrating that FLD is associated with an increased CV risk, ASA use in FLD patients seems an important consideration in clinical practice.

#### Renin Angiotensin Inhibitors

Patients with FLD usually present with an increased prevalence of several risk factors such as hypertension, type 2 diabetes mellitus and kidney disease. This indicates that the activation of the renin angiotensin system (RAS) might be playing a role leading to liver fibrosis. Several clinical trials studied the effects of antihypertensive agents in FLD patients demonstrating that hypertensive patients who received RAS antagonists had reduced levels of FLD progression (154–157). A recent observational study conducted on 118 patients with NAFLD who were followed for a median of 36 months demonstrated that diabetic patients using of RAS inhibitors such as angiotensin converting enzyme inhibitors or angiotensin receptor blockers were associated with reduced histological fibrosis progression rates (158). Nevertheless, randomized clinical trials are required to confirm this effect.

#### Lipid Lowering Medications

Despite the controversial evidence linking the use of statins and ezetimibe with a decreased risk of FLD, mainly due to grossly underpowered studies, mainly observational and not experimental studies, in addition to the lack of histological assessment of liver injury severity (159–161), they remain a key therapy for reducing the risk of adverse CV events as well as mortality (162, 163). Current studies reported that statins use was associated with a reduced CV risk (164, 165) and improved liver enzymes as demonstrated in a *post-hoc* analysis of the Greek Atorvastatin and Coronary Heart Disease Evaluation (GREACE) study (166). Statin therapy shouldn't be precluded in subjects with FLD, high CV risk and elevated aminotransferases as current evidence does not prove liver damage in this context (167).

Moreover, high dose omega-3 fatty acids were not found to be associated with improved histological features in NASH (168, 169), although some evidence suggests an improvement in dyslipidemia and a reduction in liver enzymes (170).

#### Insulin Sensitizers, Incretin Mimetics, and Other Anti-diabetic Drugs

Insulin resistance is significantly involved in the pathogenesis of FLD. Several therapeutic agents that are currently approved for treating type 2 diabetes mellitus have been evaluated for possible positive outcomes related to liver injury in patients with FLD.

Metformin remains the cornerstone therapy for type 2 diabetes mellitus patients. Despite the fact that metformin use in NAFLD and NASH patients was associated with improved insulin resistance and reduced aminotransferase levels (171), it failed to improve liver injury evaluated histologically or NASH reported in a meta-analysis (134, 156). Nevertheless, cross-sectional studies demonstrated that metformin use may protect against the progression to hepatocellular carcinoma (172), as well as a reduced mortality rate in cirrhotic patients (173). Therefore, the use of metformin should be considered in type 2 diabetic patients and FLD, unless a contraindication such as severe hepatic or renal failure is present. Moreover, a randomized double-blind placebo controlled trial performed on 68 patients with prediabetes and/or insulin resistance demonstrated that metformin treatment was significantly associated with reduced LV mass index, office systolic BP, reduced body weight and oxidative stress explaining that metformin exerts a cardioprotective effect and suggesting the possibility of metformin use in CAD patients who are non-diabetics (174).

On the other hand, alternative therapeutic drugs mainly targeting insulin resistance seem to be associated with better hepatic histological outcomes in NASH. Examples include pioglitazone, a peroxisome proliferator- activated receptor- γ (PPARγ) agonist (175, 176) and liraglutide, a glucagon- like peptide 1 (GLP1) agonist (177). The PIVENS (Pioglitazone vs. Vitamin E vs. Placebo for the Treatment of Non-diabetic Patients with Non-alcoholic Steatohepatitis) trial demonstrated that despite the histological improvement of NASH except fibrosis associated with Pioglitazone, vitamin E, a lipophilic antioxidant vitamin E was reported to be more effective in improving and resolving inflammation (175). However, a recently published study suggests that treatment with Pioglitazone for a long-term can lead to a reduction in liver damage as well as fibrosis (176). The decision of starting treatment with Pioglitazone should be considered for each patient individually based on risks and benefits due to several side effects such as causing or exacerbating congestive heart failure in some patients (178). Moreover, the GLP-1 agonist, liraglutide, was evaluated in several studies. The phase II LEAN study demonstrated a better NASH resolution rate in addition to a reduced liver fibrosis progression and weight loss (177). The LIRA-NAFLD study demonstrated that uncontrolled type 2 diabetic patients were able to achieve a reduction in their liver fat content of an average 31% after receiving liraglutide 1.2 mg/day for 6 months (179). The LEADER (Liraglutide Effect and Action in Diabetes: Evaluation of Cardiovascular Outcome Results) trial reported that type 2 diabetic patients that received liraglutide treatment presented with reduced rates of fatal and non-fatal adverse CV events as well as death from any cause compared to the placebo group (180).

Recent studies including randomized controlled clinical trials demonstrated that sodium-glucose cotransporter type-2 (SGLT2) inhibitors can improve hepatic steatosis and serum liver enzymes in type 2 diabetic patients with NAFLD as well as exert the already well-known CV protective effects (181). On the other hand, a study conducted on diabetic patients demonstrated that Sitagliptin, the DPP-4 inhibitor, was not to be effective in hepatic fat content reduction (182).

Furthermore, other drugs with an insulin sensitizing effect are currently under evaluation, such as elafibranor, a PPARα-PPARδ agonist, evaluated in phase IIB randomized placebo-controlled GOLDEN 505 trial (183) where is was demonstrated to improve serum lipid levels and liver enzymes. Currently, the overall benefit is under evaluation in the phase III RESOLVE-IT trial. Moreover, other promising novel agents such as IVA337, a PPARα-PPARδ-PPARγ agonist and saroglitazar, a PPARα-PPARγ agonist are currently being evaluated in randomized controlled clinical trials in patients with FLD in order to assess their efficacy as treatment for NASH in late phase clinical trials (184). The CV outcomes and benefits related to the use of these drugs remain to be assessed.

#### Semi-Synthetic Bile Acid Analog

Obeticholic acid, a potent agonist of Farnesoid X Receptor (FXR) with activity against hepatic fat accumulation, inflammation and fibrosis, has been studied in the phase IIb Farnesoid X Receptor Ligand Obeticholic Acid in NASH Treatment (FLINT) trial in non-cirrhotic NASH patients with results demonstrating an improvement in NASH histology with the main safety signals being elevated LDL levels despite concomitant statin use, possible worsening of insulin resistance, as well as pruritus (185). The phase III REGENERATE trial is being conducted which will also include CV risk assessment.

### Surgical Management

Bariatric surgery can be useful in a specific group of patients with fatty liver disease that are unresponsive to several trials of lifestyle changes as well as intensive pharmacotherapy as it can improve NASH histologically, lead to weight loss and reduce obesity-related metabolic complications, while demonstrating long-term stable results (10, 186). However, bariatric surgery remains an invasive procedure with possible complications and increased costs, limiting its assessment as a primary management strategy in NASH patients, currently being limited mainly to manage obesity.

## Conclusions

This review supports the existence of a strong association between the presence and severity of FLD leading to several CV adverse events such as CAD, subclinical atherosclerosis risk, structural and functional cardiac modifications in addition to cardiac arrhythmias and conduction defects. Currently, the most common cause of death in NAFLD is CVD.

Despite the current evidence supporting the association between FLD and an increased CV risk, the exact mechanisms attributing to this relationship remain uncertain. Therefore, further research is required in order to discover the exact pathophysiological mechanisms behind this association to prevent further complications.

The current data emphasizes how FLD is a multisystem disease by presenting adverse effects on the liver in addition to extrahepatic manifestations including cardiac and vascular complications. This also entails that FLD patients with a high CVD risk should undergo a careful CV screening regardless of the presence of the traditional CV risk factors as per the current practice recommendations by the European Association for the Study of the Liver (EASL), European Association for the Study of Diabetes (EASD) and European Association for the Study of Obesity (EASO) as well as the American Association for the Study of Liver Diseases.

Accordingly, a multidisciplinary patient centered, personalized medicine approach is required to provide FLD patients with the best management. Encouraging FLD patients to undergo early lifestyle changes such as weight loss, exercise and dietary changes is highly recommended. Other possible pharmacological therapies include anti-diabetic drugs such as metformin, SGLT2 inhibitors, PPARγ and GLP-1 agonists, lipid lowering medications mainly statins as well as renin angiotensin system inhibitors due to their effect in reducing cardiovascular risk especially in the presence of dyslipidemia, diabetes and hypertension. Surgical management remains of limited use due to its invasiveness, high costs and possible complications.

## Author Contributions

AI had the idea of this review, did the literature survey and wrote the manuscript. DD checked the literature survey, suggested methodology and contributed to the writing of the manuscript. All authors revised the final manuscript and approved the final version.

### Conflict of Interest Statement

The authors declare that the research was conducted in the absence of any commercial or financial relationships that could be construed as a potential conflict of interest.

## Abbreviations

AF: Atrial Fibrillation
AFL: Alcoholic Fatty Liver
AFLD: Alcoholic fatty liver disease
ALD: Alcoholic Liver Disease
ALT: Alanine transaminase
ASH: Alcoholic Steatohepatitis
BMI: Body Mass Index
CAC: Coronary Artery Calcium
CAD: Coronary Artery Disease
CIMT: Carotid Intima Media Thickness
CLD: Chronic Liver Disease
CRP: C-reactive Protein
CV: Cardiovascular
CVD: Cardiovascular Disease
EFT: Epicardial Fat Thickness
FLD: Fatty liver disease
GGT: Gamma-glutamyltransferase
HbA1c: Glycated hemoglobin
HDL: High Density Lipoproteins
IR: Insulin Resistance
LV: Left Ventricular
MetS: Metabolic Syndrome
NAFL: Non-alcoholic Fatty Liver
NAFLD: Non-alcoholic Fatty Liver Disease
NASH: Non-alcoholic steatohepatitis
VAT: Visceral Adipose Tissue.
